# The prognostic value of simultaneous tumor and serum RAS/RAF mutations in localized colon cancer

**DOI:** 10.1002/cam4.1051

**Published:** 2017-04-04

**Authors:** Caroline Emilie B. Thomsen, Ane Lindegaard Appelt, Rikke Fredslund Andersen, Jan Lindebjerg, Lars Henrik Jensen, Anders Jakobsen

**Affiliations:** ^1^ Danish Colorectal Cancer Center SouthVejle HospitalBeridderbakken 4VejleDK‐7100Denmark; ^2^ Institute of Regional Health ResearchUniversity of Southern DenmarkWinsløwparken 19‐3OdenseDK‐5000Denmark; ^3^ Department of Radiation OncologyRigshospitaletBlegdamsvej 9Copenhagen EastDK‐2100Denmark; ^4^ Leeds Institute of Cancer and PathologyUniversity of Leeds and St James's University HospitalLeedsUnited Kingdom

**Keywords:** BRAF, colon cancer, liquid biopsy, RAS, serum

## Abstract

The impact of RAS/RAF mutations in localized colon cancer needs clarification. Based on analysis of tumor‐specific DNA, this study aimed at elucidating the prognostic influence of mutational status in tumor and serum using an extended panel of mutations.

The study retrospectively included 294 patients with curatively resected stage I–III adenocarcinoma of the colon. Mutations in tumor and serum were determined at time of surgery. Analyses were performed with droplet digital PCR technology. Hazard ratio (HR) for the association between mutational status and survival was estimated in multivariate analysis taking known prognostic factors into account.

Mutational status in tumor did not on its own have significant prognostic impact (*P* = 0.22). Patients with a RAS mutation simultaneously in tumor and serum had a significantly worse prognosis, overall survival (OS) (HR = 2.30, 95% CI = 1.27–4.15, *P* = 0.0057), and disease‐free survival (DFS) (HR = 2.18, 95%CI = 1.26–3.77, *P* = 0.0053). BRAF mutation in the serum and proficient mismatch repair (pMMR) protein in tumor also indicated significantly worse prognosis, OS (HR = 3.45, 95% CI = 1.52–7.85, *P* = 0.0032) and DFS (HR = 3.61, 95% CI = 1.70–7.67, *P* = 0.0008). In conclusion, RAS mutations in serum, and BRAF mutation in serum combined with pMMR in tumor were strong independent prognostic factors in patients with RAS/RAF mutated tumors.

## Introduction

Malignant tumors are generally characterized by somatic mutations. It is assumed that mutations develop over time and that the pattern may well be different in early and late stages. However, a new hypothesis suggests that most mutations occur in the early phase and that occurrence of late mutations is not likely to result in the development of new clones 1. Irrespective of the time course of development, somatic mutations represent important biological characteristics likely to influence the behavior of the tumor.

RAS/RAF mutations are early events in the development of colorectal cancer. The RAS/RAF proteins affect gene expression by encoding GTP‐binding proteins, which act as molecular switches connecting extracellular signals with nuclear transcription factors. Mutations in RAS/RAF result in a constitutive activation of the Ras/Raf/Mek/Erk/Map pathway independent of the stimulation of the epidermal growth factor receptor (EGFR). This cascade facilitates growth and proliferation leading to migration and invasion 2.

RAS mutations are point mutations most frequently found in the KRAS and NRAS genes. Patients with metastatic colorectal cancer harboring RAS mutations do not benefit from monoclonal antibodies blocking the EGFR 3 and may even have a detrimental effect from treatment with EGFR‐inhibitors 4. Thus, these mutations occurring in approximately 50% of colon cancers have considerable clinical interest. Mutation analysis performed on tissue samples is standard practice before start of EGFR‐inhibitor treatment. However, while the predictive value of RAS mutations as to the benefit of EGFR‐inhibitor treatment is well established, the overall prognostic importance of RAS mutations is not clear. Most studies on the issue include both colon‐ and rectal tumors, and a possible difference in prognostic value in the two groups cannot be extracted from the literature. It is also worth noting that almost all studies include only metastatic disease or a mixture of metastatic and localized disease, likely due to the predictive value of RAS mutations in relation to biologically targeted treatment in metastatic disease.

The most frequent BRAF mutation is a GTG>GAG substitution at position 1799 of exon 15, which results in the V600E amino acid change. BRAF mutations occur in 8–10% of metastatic colorectal cancers but are reported in 10–22% of localized colon tumors. The BRAF mutation holds prognostic information in metastatic disease but its potential importance in localized colon cancer remains to be proven. Recent studies have not reported any significant independent prognostic value of BRAF mutations 5, 6, 7, but mismatch repair (MMR) status may be an effect modifier 8. A subset of BRAF mutated colorectal cancers is characterized by high levels of microsatellite instability (MSI‐H). The underlying mechanism is methylation of the MLH1 promotor, which blocks the transcription of mismatch repair protein MLH1. Immunohistochemically, these tumors show concurrent loss of MSH1 and PMS2. Approximately 40% of tumors with methylator inactivation of MLH1 harbor a BRAF mutation. It is possible that MLH1/PMS2 status modifies the prognostic effect of a BRAF mutation.

So far, most studies on the impact of somatic mutations have been based on tumor tissue analyses with divergent results. Peripheral blood (plasma and serum) may provide a better overall reflection of the status as reported in an increasing number of studies 9, 10, 11.

Adjuvant chemotherapy benefits only a minor subgroup of colon cancer patients and a better selection is of utmost importance. New reliable, prognostic markers suitable for clinical application may serve an important role for better classification of patients for adjuvant chemotherapy or close observation.

This study aimed at elucidating the prognostic importance of RAS/RAF mutations in localized colon cancer extending the panel to 27 mutations. Furthermore, the additional prognostic value of mutated DNA in serum of patients with a tumor mutation was analyzed.

## Materials and Methods

### Patients

This retrospective study cohort consisted of 294 patients who underwent surgery for colon cancer at Vejle Hospital between January 1, 2010 and December 31, 2013. The inclusion criteria were complete resection (except for 6 patients (2%) with microscopic involvement of the surgical margin), adenocarcinoma, and pTNM stage I‐III. Adjuvant treatment was given to patients with high‐risk stage II and stage III (*n* = 51, 17.4%) according to international guidelines 12. Patients not receiving adjuvant chemotherapy and those receiving adjuvant treatment without complications (*n* = 270, 91.8%) were followed with clinical examination and a chest and abdominal CT scan one and 3 years after the surgery, and then every 5 years with colonoscopies until the age of 75. Patients with complications during the adjuvant treatment (*n* = 24, 8.2%) underwent a follow‐up program with chest and abdominal CT scan and clinical examination every 3 months the first year, every 6 months the second and third year, and then yearly the fourth and fifth year.

The study was approved by the Ethics Committee for Southern Denmark (S‐20140178). The investigation was conducted in accordance with the REMARK criteria 13.

### Pathology

Tumor tissue was formalin‐fixed and paraffin embedded (FFPE) by routine methods and all samples were stored under standard and consistent conditions. All tumors were staged I–III according to UICC and stained by immunohistochemistry for MLH1, PMS2, MSH2, and MSH6. Based on the staining results, patients were divided into three groups: (1) Proficient MMR (pMMR), (2) Loss of MLH1/PMS2 only (deficient, dMLH1/PMS2 only) and (3) Otherwise abnormal MMR expression (dMMR others).

### Tumor sample mutation analysis

Droplet Digital polymerase chain reaction (ddPCR) (BioRad, Hercules, CA) was used for the analysis of all mutations in both tissue and serum as described below.

The investigation included 27 mutations with a frequency >0.2% in colorectal cancer. The mutations were selected by combining the results from two previous studies 4, 14. The analyses were performed in three rounds. We tested the three most frequent KRAS mutations (codon 12: G12D and G12V and codon 13: G13D) and the BRAF mutation (V600E) in the first round, and negative samples were tested for six mutations occurring less frequently in the second round. Leaving out the negative ones, the rest of the panel (17 mutations) was tested in the third round (Table S1).

Tumor slides were reviewed for selection of paraffin blocks with abundant tumor cells. Three 15 *μ*m slices of FFPE tumor tissue were subjected to 180 *μ*L) incubation buffer and 20 *μ*L protein kinase K overnight at 70°C. Four hundred *μ*l lysis buffer was added to the DNA samples that were purified on the MAXWELL^TM^ 16 LEV instrument using FFPE Plus LEV DNA Purification kit (Promega AS1135) according to the manufacturer's recommendations. DNA was eluted in 50 *μ*L nuclease‐free water and further diluted with 500 *μ*L nuclease‐free water. BioRad ddPCR supermix, PrimePCR ddPCR assays for specific mutations (Bio‐Rad^®^, Table S2) and purified DNA were mixed with oil and droplets were generated in the Automated Droplet Generator (Bio‐Rad^®^). For multiplex reactions, wild‐type assays and assays for each mutation were mixed in equal amounts and 2 *μ*L was used for each 20 *μ*L reaction. Forty cycles of PCR amplification were carried out (initial denaturation at 95°C for 10 min, 40 cycles of 94°C for 30 sec and 55°C for 60 sec, and final extension at 98°C for 10 min), and the samples were analyzed for droplets containing mutated and wild‐type DNA in the Droplet Reader QX100 (Bio‐Rad^®^). Quantasoft ddPCR software ver. 1.7 was used for analyzing data. Data were visualized and the concentrations of droplets with a mutation were quantified.

Patients presenting with a RAS/RAF mutation in the tumor were selected for serum screening. Based on the mutation detected in primary tumor tissue, the blood was screened for the specific mutation and analyses were performed as described below.

### Blood sample mutational analysis

Blood samples were collected at time of surgery (within a 4‐day window prior to the operation). A 9 mL peripheral blood sample was collected and left to coagulate for at least 30 min. Serum was isolated by centrifugation at 2000*g* for 10 min within 2 h after collection and stored at −80°C. After defrosting the serum was centrifuged at 10,000*g* for 10 min. Cysteine‐rich polycomb‐like protein1 (CPP1) DNA fragments were added as exogenous internal control before purification 15. DNA was extracted from 4 mL serum with the MagnaPure MPLC Total NA Isolation Kit‐Large Volume (Roche, Basel, Switzerland) and water was added to the samples with inadequate volume prior to purification. DNA was eluted in 400 *μ*L. qPCR was performed to quantify total cell‐free DNA (cfDNA) and CPP1 as previously shown 15. The remaining DNA (380 *μ*L) was concentrated to 20 *μ*L on a Millipore centrifugal filter unit (Millipore, Billerica, MA). DNA was preamplified 10 cycles with Q5 mastermix (New England Biolabs) and PrimePCR ddPCR assay (diluted to 0.11 × final concentration) for the specific mutation (Bio‐Rad^®^) in 50 *μ*L reaction volumes according to the manufacturer's recommendations. Negative controls (water and donor DNA) and positive controls were preamplified along with the samples and analyzed as the samples. Positive controls for all mutations were generated using site‐directed mutagenesis as described previously 16. DNA was diluted 50–400 times prior to ddPCR analysis based on the cfDNA qPCR quantification. 5 *μ*L of DNA was combined with PrimePCR ddPCR assays for specific mutations (Bio‐Rad^®^) and Bio‐Rad^®^ ddPCR supermix in reactions of 20 *μ*L for PCR amplification as described above. Water and positive controls were included in each ddPCR. These were all mixed with oil and droplets were generated in an Automated Droplet Generator (Bio‐Rad^®^). All samples were analyzed in duplicate. Forty cycles of PCR amplification were carried out, and the fluorescence signals were analyzed in a Droplet Reader QX100 (Bio‐Rad^®^) for droplets positive for FAM or HEX signal corresponding to mutated or wild‐type DNA. Quantasoft ddPCR Software ver. 1.7 was used for analyzing data, data were visualized, and the concentrations of droplets with mutated or wild‐type DNA were quantified.

### Data analysis

Limit of blank (LOB) of all mutation detection assays was determined on preamplified donor DNA analyzed together with the samples. LOB varied among the assays from 0 to 0.06% (0–23 copies/well) analyzing a minimum of 2 × 30,000 wild‐type copies. From merged data of double measurement, Quantasoft ddPCR Software calculated the fraction of mutated alleles/total alleles in patient samples. To define a positive sample, the 95% confidence interval (95% CI) of the measurements for the donor and the sample had to be separate (nonoverlapping). The interpretation of the results was identical for RAS and BRAF mutations 17.

Results were analyzed using NCSS 10 Statistical Software (2015) (NCSS, LLC. Kaysville, Utah, USA, ncss.com/software/ncss) and R version 3.2.2 (R Foundation for Statistical Computing, Vienna, Austria, 2015). The primary outcome measure was overall survival (OS), defined as the time from operation to death from any cause. Data on cause and date of death were obtained from the Danish Civil Registration System. The secondary outcome measure was disease‐free survival (DFS), defined as the time from operation to the first documented recurrence, locally or distant, of colon cancer or death of any cause. Date of recurrence was defined as the date of physician‐determined recurrence in the clinical notes based on radiography, biopsy, or multidisciplinary team conference. Patients were censured for recurrence at the date of their most recent disease evaluation or hospital contact. The occurrence of secondary cancer did not led to censoring of data. Survival analyses were carried out using the Kaplan–Meier estimator, and survival curves for subgroups were compared using the log‐rank test. The independent prognostic value of mutation status in tissue and serum was examined by multivariate proportional hazard Cox regression, taking known clinical risk factors (stage, differentiation, neural‐ or vascular involvement, and perforation of the peritoneum) into account. Validity of the proportional hazard assumption was ensured by testing for correlation between Schoenfeld residuals and time. To compare the impact of mutation status in tissue and serum, respectively, on the prognostic performance of the Cox regression models, concordance indices (c‐indices) were calculated and compared for each of the regression models. Additionally, the prognostic influence of variations in the quantitative mutation load was examined for patients with serum mutations, taking known risk factors into account and modeling the mutation load dependence with a restricted cubic spline function. All reported *P*‐values were two‐sided and *P* < 0.05 were considered statistically significant.

## Results

Tumor tissue for mutational analyses was obtained from 293/294 patients (99.7%) and mutation status in the serum was analyzed in 187/189 patients (98.9%) with two samples lost because of error in the analyses. Patient characteristics are shown in Table 1. pMMR status was found in 73.8% of the patients, whereas 21.1% had dMLH1/PMS2 only, and 5.1% had other dMMR. A RAS/BRAF tumor mutation was detected in 64.5%, and mutated DNA was found in the serum of 42.3% of these patients.

**Table 1:** Baseline patient characteristics (*n* = 294)

	*n* (%)
Median age, year (range)	73 (35–94)
Gender	
Female	150 (51)
Male	144 (49)
pT‐category	
T1	9 (3)
T2	10 (3)
T3	11 (3)
T4	12 (3)
Stage	
I	40 (14)
II	151 (51)
III	103 (35)
Differentiation grade	
High	246 (84)
Low	26 (9)
Mucinous	22 (7)
MMR	
pMMR	217 (74)
dMLH1/PMS2 only	62 (21)
dMMR others	15 (5)
Tissue	
Mutated	189 (65)
Wt	104 (35)
ND	1 (0.3)
Serum	
Mutated	80 (42)
Wt	107 (56)
ND	2 (1)

*n*, number; pT‐category, pathologic Tumor stage; MMR, mismatch repair; pMMR, proficient MMR; dMMR, deficient MMR; Wt, wild type; ND, not determined.

### Mutational status

The mutational status is further detailed in Table 2. It should be noted that the most frequent KRAS mutations in codon 12 and 13 were less frequent in the serum compared to the BRAF mutation. Approximately half of the tumors with NRAS or BRAF mutation had mutated DNA detectable in the serum. A complete list of mutations is given in the supplementary material (Table S3). Detection of mutations in the serum varied according to stage (Table S4). Seventeen percent (4/24) of patients with stage I disease and a mutation in tumor also had a RAS/BRAF mutation in the serum. The rate increased to 42.9% and 51.7% in stage II and III patients, respectively.

**Table 2:** Frequency of mutations in tumor and serum

	Wild type	Mutations in tissue (%)	Mutations in serum (%)
KRAS Codon 12 + 13	195	98/293 (33.4)	29/98 (29.6)
KRAS Codon 61 + 117 + 146	284	9/293 (3.1)	4/9 (44.4)
NRAS Codon 12 + 13 + 61	287	6/293 (2.0)	3/6 (50.0)
BRAF V600E	217	76/293 (25.9)	44/76 (57.9)
ND	—	1	3

ND, not determined.

Mutation load, expressed as the proportion of mutant alleles in cfDNA (mutant and wild‐type alleles), was highly variable among mutant samples (0.005–11%), median = 0.098%. Further details are given in the supplementary material (Table S4).

### Survival analysis

The clinical database was last updated on October 1, 2015. Median follow‐up time for OS was 3.9 years and for DFS 3.0 years as estimated by inverse Kaplan–Meier analysis. The OS analysis was based on 73 events and the DFS analysis on 87 events among 294 patients.

**Figure 1:**
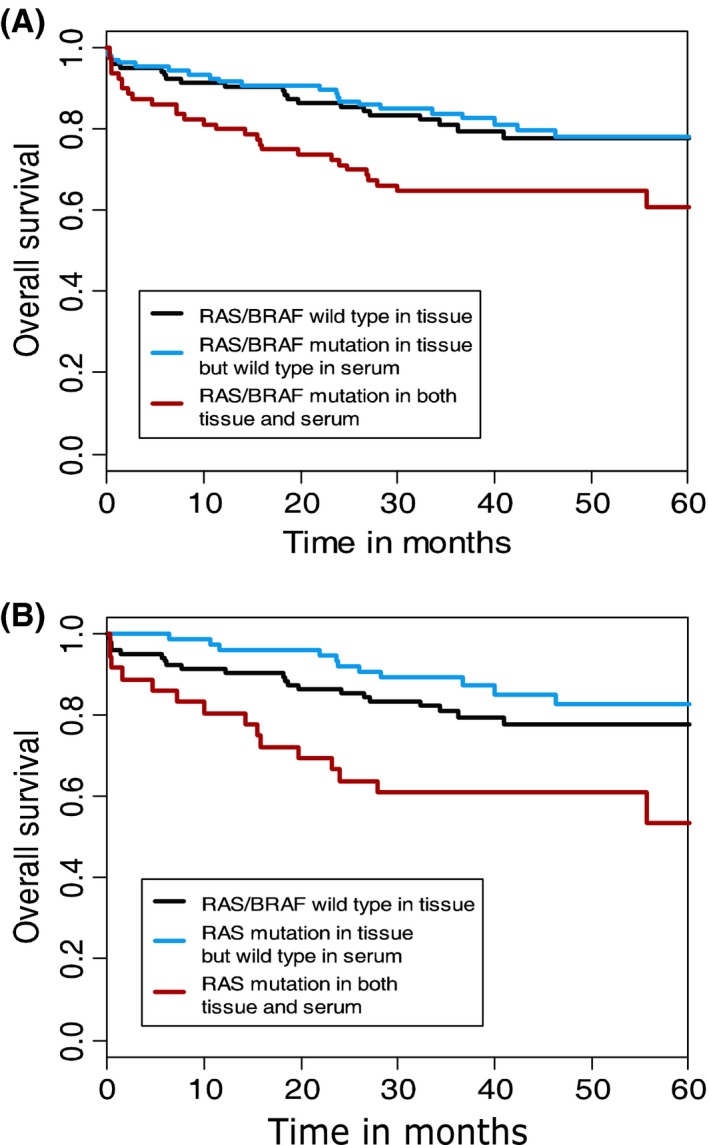
Overall survival. **(**A) OS according to mutational status. Three‐year survival rates were 81%, 84%, and 65%, respectively. (B) OS according to RAS mutational status. Three‐year survival rates where 81%, 89%, and 61%, respectively.

Overall survival according to mutational status in tumor and serum is shown in Figure 1A. There was no clinical or significant difference in risk of event between patients with a wild‐type tumor and those with a RAS/BRAF mutation in the tumor (*P* = 0.22), and the same was true as to DFS (*P* = 0.31). The results remained nonsignificant when subdividing patients according to RAS and BRAF mutations. There was no significant prognostic influence of MMR status when dividing into the three groups (pMMR, dMLH1/PMS2 only, and dMMR others).

**Figure 2:**
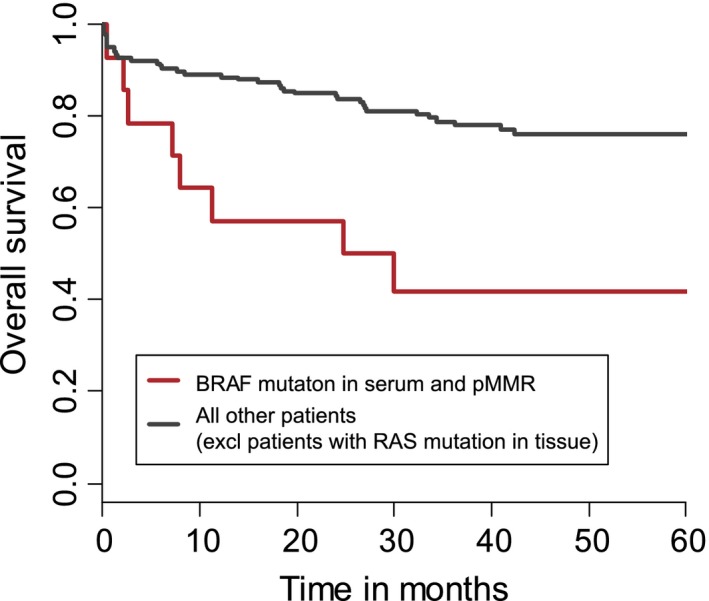
Overall survival for BRAF mutation in serum and pMMR in tumor. OS according to BRAF mutational status in the serum and MMR status in the tumor. Three‐year OS rates were 79% and 42%, respectively.

The analyses demonstrated a significant correlation between serum mutations and prognosis. A RAS or BRAF mutation both in the serum and in the tumor resulted in significantly worse OS (*P* = 0.012) and DFS (*P* = 0.026) compared to a mutation in the tumor only. The effect of a RAS mutation alone is shown in Figure 1B, which compares patients with RAS/BRAF wild‐type tumors with patients harboring a RAS mutation in tissue only and patients with a RAS mutation in tissue and serum. The 3‐year survival rates differed significantly (89% and 61%, *P* = 0.0012) between patients with a mutation in tumor only compared to patients with a mutation in both tumor *and* serum. Similar analyses on the effect of a BRAF mutation showed a OS of, respectively, 71% and 68% for BRAF mutation in tumor alone and in tumor and serum (*P* = 0.96). The combination of BRAF mutated DNA in the serum from patients with pMMR tumors, however, indicated a worse prognosis (*P* = 0.0007) as shown in figure 2.

The conventional prognostic factors of OS were analyzed in a univariate analysis. Stage I compared to stage III disease had the expected effect, HR (stage I/III) 0.50, 95% CI: 0.25–0.99, although not significant (*P* = 0.090). Neural‐ or vascular involvement and perforation of the peritoneum were highly significant, *P* = 0.0051 and *P* = 0.0025, respectively. Differentiation grade (high vs. low and mucinous combined) was nonsignificant (*P* = 0.47).

To investigate the prognostic value of the mutations in the context of conventional prognostic factors, a multivariate Cox regression analysis included the factors (1) RAS mutation detected in both tissue and serum, and (2) BRAF mutation in serum combined with pMMR status in tumor, in addition to known prognostic factors. BRAF mutation in serum and pMMR individually were not tested because of the insignificant result in the univariate analysis. Adjuvant therapy and number of affected lymph nodes were not included in the model because of collinearity with the other factors. The results are shown in Table 3.

**Table 3:** Cox regression analysis

	OS HR (95% CI)	DFS HR (95% CI)
RAS mutation in serum	2.30 (1.27–4.15)	2.18 (1.26–3.77)
BRAF mutation in serum and pMMR in tumor	3.45 (1.52–7.85)	3.61 (1.70–7.67)
Stage		
I vs. II	1.13 (0.50–2.59)	1.16 (0.54–2.50)
I vs. III	1.33 (0.56–3.16)	1.28 (0.57–2.89)
Differentiation		
High vs. low	0.99 (0.43–2.31)	0.91 (0.42–1.99)
High vs. mucinous	1.41 (0.59–3.38)	1.17 (0.50–2.72)
Neural‐ or vascular involvement	1.48 (0.84–2.59)	1.58 (0.94–2.66)
Perforation of the peritoneum	2.10 (1.09–4.04)	1.87 (1.02–3.45)

The analysis is based on all patients with full data (*n* = 291).

Harboring detectable RAS mutated DNA in the serum remained an independent significant prognostic factor both for OS (HR = 2.30, 95% CI = 1.27–4.15, *P* = 0.0057) and DFS (HR = 2.18, 95%CI = 1.26–3.77, *P* = 0.0053). The same applied to BRAF mutation in serum and pMMR in tumor. These characteristics were independently related to worse OS (HR = 3.45, 95% CI = 1.52–7.85, *P* = 0.0032) as well as DFS (HR = 3.61, 95%CI = 1.70–7.67, *P* = 0.0008). Perforation of the peritoneum also remained significant in the multivariate analysis. A comparable model based on tissue mutations and MMR status (including the same standard prognostic factors) did not show any impact of mutation status on OS and DFS. The c‐index for the Cox model taking serum mutation status into account was 0.67 (95% CI = 0.60–0.74), which was significantly better than the c‐index for the model using tissue mutation status (*P* = 0.01).

All factors except for neural‐ or vascular involvement met the proportional hazard assumption as did the model in general. A robustness analysis including neural‐ or vascular involvement as a stratification factor rather than as a model parameter resulted in almost identical hazard ratios and *P*‐values for the impact of mutational status.

As an illustration of the prognostic value of RAS mutation in the serum, we estimated the 3‐year OS of a standard patient (stage II disease, highly differentiated tumor, no perforation of peritoneum, no neural‐ or vascular involvement, and no BRAF mutation) based on the multivariate Cox regression analysis. The estimated 3‐year OS for such a patient *without* a RAS mutation in the serum was 86% (95% CI = 80–92%) and *with* a RAS mutation 70% (95% CI = 57–87%). This is different from the model based on RAS mutational status in tissue: 84% (95% CI = 78–92%) without RAS mutation and 83% (95% CI = 76–91%) with RAS mutation. See supplementary material for graphical illustration of the estimated survival curves with/without RAS mutations, taking other prognostic factors into account (Figs. [Link], [Link], [Link], [Link]).

**Figure 3:**
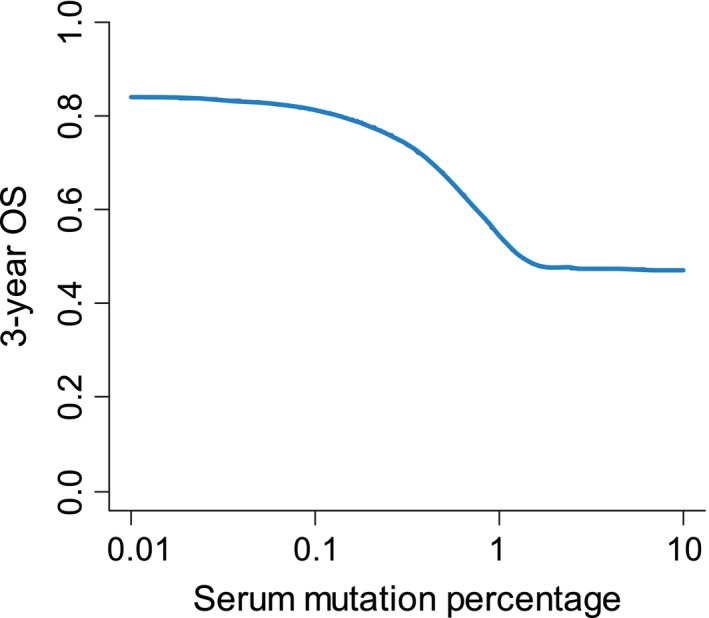
Overall survival for quantitative mutation load. An illustration of the dependence of 3‐year OS on the quantitative measurement of the mutation load. NOTE: Because of the broad range of values, the *x*‐axis is logarithmic.

The mutational load also held prognostic information. Figure 3 illustrates the 3‐year survival rate based on Cox regression analysis with conventional prognostic factors and mutation load as continuous variables. The effect on survival appears most marked in the interval 0.1–1.0% mutated alleles.

## Discussion

Colon cancer represents a major therapeutic challenge. Surgery is a cornerstone in the standard treatment strategy, but is not always curative, especially in patients with stage III disease. Additionally, adjuvant chemotherapy has shortcomings. It provides a minor improvement in overall survival but may have serious side effects. These challenges call for proper selection of patients, and the generally accepted criteria 12 are inadequate. Consequently, new prognostic markers have high priority, especially in patients with stage II disease.

Mutations are important to the biology of colon cancer and the RAS and RAF mutations in tumor tissue have been extensively investigated as predictive and prognostic markers, particularly in the metastatic setting. Routine assessment of the RAS mutational status is performed to personalize treatment of metastatic disease, and the panel of mutations analyzed has expanded through the years.

At present, there is no consensus on the prognostic impact of RAS/RAF mutations in localized disease, but it is noteworthy that almost all studies are based on analyses of KRAS codon 12 and 13 together with BRAF V600E (a total of eight mutations). This study aimed at investigating whether the contradictory results in the literature could be explained by a too narrow panel of mutations. The results, however, did not suggest any prognostic importance of an expansion toward a broader panel of analyzed mutations. Our results are in agreement with a study by Roth et al. 18 who analyzed 1404 stage I–III colon cancer patients and found a frequency of KRAS mutations similar to our results but no prognostic impact on OS (*P* = 0.45). On the other hand, a study with 1989 patients concluded that KRAS mutation was associated with worse survival 19. This investigation, however, differs from ours in several aspects. It included both rectal‐ (33%) and colon cancer and metastatic disease (11%). It should also be noted that a stratified analysis by stage showed no significant influence of KRAS mutations on the OS in localized disease (HR = 1.19, 95%CI = 0.87–1.63) and 29% of the patients had missing information on KRAS status.

BRAF mutations are anticipated to have prognostic impact in metastatic colorectal cancer 20, 21, but their importance in localized disease is dubious. A study with 364 stage II and III colon cancer patients reported a poor prognosis for patients with BRAF mutations in tumor independent of age, stage, grade, differentiation, MMR status, and location 22. It is notable that 71% of the patients received adjuvant chemotherapy, which is a large fraction of an average population with stage II–III disease, hampering comparison with our study where 17% received adjuvant chemotherapy. Previous reported results 23, 24 and a recent review point to a prognostic impact of BRAF mutations together with MMR status and location 8. Our results confirmed this finding. The proportion of sporadic dMMR cancers (group B) is similar to what is found in the background population. MMR status was divided into three groups to separate sporadic cancer and dMMR from patients with possible Lynch Syndrome.

Analysis of serum mutations calls for high sensitivity, especially in localized disease with low tumor load. ddPCR has a sensitivity of around 10^−4^ as also found in our study. An important factor is the LOB, which sets the lower level of mutation detection in the current sample 25. We classified a sample as positive if the value was above the upper level of the 95%CI of the negative control samples. Plasma is probably preferable to serum for mutation analysis because of contaminating wild‐type DNA in serum 26. We used serum, since plasma was not available and the low LOB allowed for a high sensitivity, but mutations occurring at very low frequencies cannot be excluded. On the other hand, mutations at very low frequencies (<0.1%) only seem to have marginal prognostic influence (Fig. 3).

The presence of a RAS mutation in the serum had a significant prognostic value, both in the univariate and multivariate analysis. The literature on this issue is sparse and the results are difficult to interpret because of different methodologies and patient materials. The majority of the studies included both colon‐ and rectal cancer, and it is well known that the frequency of RAS/RAF mutation differs with the location of the tumor. Additionally, most studies included localized as well as metastatic disease and were based on small numbers of patients as described in a recent review 27. The first prospective study from 2003 found no prognostic impact on disease recurrence of KRAS mutation in the serum preoperatively. Despite a high relative hazard of 2.07 the difference was not significant (95%CI = 0.3–14.8, *P* = 0.466) 9. The study did not report on OS. The authors found a much higher frequency of KRAS mutations (codon 12 and 13) in the serum, provided that the tumor was KRAS mutated, 76% as opposed to 30% in this study. The trial differs from our study in many important aspects. It was a smaller study than ours (*n* = 32/78 with a KRAS mutation in the serum) and they had a more inhomogeneous cohort including rectal cancer (50%) and metastatic disease (14%). Follow‐up was only reported for 49 patients (63%) and median follow‐up was not stated. Furthermore, they analyzed for the seven most frequent KRAS mutations (codon 12 and 13).

We found that the frequency of BRAF mutations in the blood was almost doubled compared to that of RAS (58% vs. 32%). BRAF mutation in the serum alone was not significant as a prognostic factor. In agreement with the theory that MMR status links prognosis with BRAF mutational status, we found a statistically significant negative prognostic value of BRAF mutation in the serum from patients with pMMR in tumor. The independent prognostic influence was confirmed in the Cox regression analysis. To our knowledge our data are the first to indicate a prognostic relation between BRAF mutation status in the blood and MMR status in the tumor.

In the multivariate analysis we tested the prognostic value of RAS mutation in the serum and the combination of BRAF mutation in the serum and pMMR in the tumor against conventional prognostic factors and they both remained statistically significant. They came out stronger than the indicators used conventionally. The independent value was shown in the Cox regression analysis but should be considered with caution because of the wide confidence limits. The c‐index indicated that the prognostic model using serum status performed significantly better than a model using tissue mutation status.

This study provides new information on different aspects of RAS/BRAF mutations. The results failed to show any impact of mutations in tumor tissue even using an expanded panel of mutations screened for, which to the best of our knowledge has not been reported previously. The analysis of tumor‐specific DNA in serum holds new, original information. The same applies to the correlation between OS and the quantitative load of mutations. The study opens for the potential use of analysis of RAS mutations in serum in the clinical management of colon cancer, together with a combination of BRAF mutations in serum and tumor MMR status. Neoadjuvant chemotherapy may be an option in the near future and plasma mutations may serve as a tool for relevant selection. Further prognostic subclassification seems to be possible by quantification of the mutational load as recently suggested 28. A similar result was found in this study.

There are, however, limitations of the study. First of all, its retrospective nature calls for prospective validation. Therefore, our study can only be considered hypothesis generating. The study only investigated RAS/RAF mutations, and other mutations with prognostic value were disregarded but this fact does not diminish our main finding of the prognostic influence of serum mutations in RAS/RAF mutated tumors. Additionally, the subgroup of patients with BRAF mutation in serum and pMMR in tumor was small (*n* = 14). A number of additional prognostic factors could have been considered for the multivariate analysis. It is generally accepted that tumor location has a role in the prognostic impact of BRAF mutations. According to the literature the subgroup of left‐sided tumors with pMMR, and BRAF mutation in the *tissue* have the worst prognosis 8. However, location was not included in our analysis because this subgroup was very small (*n* = 3) and pMMR, left‐sided tumors and BRAF mutation in the *serum* even smaller (*n* = 1). It could be argued that CEA in the blood should be considered as well but CEA is not a validated prognostic marker according to the ESMO guidelines 12. A further limitation is the different follow‐up according to the course of adjuvant treatment, which will influence the estimate for disease‐free survival, although this subgroup was small (8%).

In conclusion, this study demonstrates a considerable clinical importance of mutations in the serum and underlines the value of “liquid biopsy”. These results emphasize the perspectives of applicating this method in the daily clinical routine but should be further developed and validated in prospective studies.

## Conflict of Interest

No potential conflicts of interest were disclosed and no financial support was given to any of the authors in relation to this work.

## Supporting information


**Figure S1.** OS with/without RAS in tissue, based on Cox regression.

**Figure S2.** DFS with/without RAS in tissue, based on Cox regression.

**Figure S3.** OS with/without RAS in serum, based on Cox regression.

**Figure S4.** DFS with/without RAS in serum, based on Cox regression.

**Table S1.** The three rounds of mutation testing.
**Table S2.** BioRad PrimePCR assays.
**Table S3**. Frequencies of the specific mutations in the cohort.
**Table S4**. Mutational load divided into quartiles and distribution in disease stages.
