# Supplementary material for: The prognostic value of simultaneous tumor and serum RAS/RAF mutations in localized colon cancer
Source: Cancer Med. 2017 Apr 4;6(5):928–36. doi: 10.1002/cam4.1051 (PMC5430097; doi:10.1002/cam4.1051)
Supplement: Supplementary file 5 — Table S1. The three rounds of mutation testing. Table S2. BioRad PrimePCR assays. Table S3. Frequencies of the specific mutations in the cohort. Table S4. Mutational load divided into quartiles and distribution in disease stages. [file CAM4-6-928-s005.doc]

**Supplementary figure legends**

Outcomes with and without RAS mutation as estimated from Cox regression, keeping the following factors constant; high differentiation, no perforation of the peritoneum, dMMR, no neural or vascular invasion, stage II, no BRAF mutation in serum combined with pMMR in tissue, and BRAF wild type in serum.

Figure S1: OS with/without RAS in tissue, based on Cox regression.

Black line: Three-year OS without a mutation in tissue: 85%, 95%CI=78-92%.

Red line: Three-year OS with a mutation in tissue: 83%, 95%CI=76-91%.

Figure S2: DFS with/without RAS in tissue, based on Cox regression.

Black line: Three-year DFS without a mutation in tissue: 79%, 95%CI=71-88%.

Red line: Three-year DFS with a mutation in tissue: 75%, 95%CI=67-85%.

Figure S3: OS with/without RAS in serum, based on Cox regression.

Black line: Three-year OS without a mutation in serum: 86%, 95%CI=80-92%.

Red line: Three-year OS with a mutation in serum: 70%, 95%CI=57-87%.

Figure S4: DFS with/without RAS in serum, based on Cox regression.

Black line: Three-year DFS without a mutation in serum: 79%, 95%CI=73-87%.

Red line: Three-year DFS with a mutation in serum: 61%, 95%CI=46-80%.

**Supplementary table legends**

Table S1: The three rounds of mutation testing.

Table S2: BioRad PrimePCR assays.

Validation data including MIQE context sequences can be found on [www.bio-rad.com](http://www.bio-rad.com/).

Table S3: TTabelT

Frequencies of the specific mutations in the cohort.

Table S4: Mutational load divided into quartiles and distribution in disease stages.

**Supplementary tables**

**Table S1:**

| **1st round** |  |  |  | **2nd round** |  |  |  | **3rd round** |  |  |  |
| --- | --- | --- | --- | --- | --- | --- | --- | --- | --- | --- | --- |
|  |  |  |  |  |  |  |  |  |  |  |  |
| **KRAS** | **Exon 2** | G12D |  | **KRAS** | **Exon 2** | G12A |  | **KRAS** | **Exon 3** | Q61H A>C |  |
|  |  |  |  |  |  |  |  | **KRAS** | **Exon 3** | Q61H A>T |  |
| **KRAS** | **Exon 2** | G12V |  | **KRAS** | **Exon 2** | G12C |  | **KRAS** | **Exon 3** | Q61R |  |
|  |  |  |  |  |  |  |  | **KRAS** | **Exon 3** | Q61L |  |
| **KRAS** | **Exon 2** | G13D |  | **KRAS** | **Exon 2** | G12S |  |  |  |  |  |
|  |  |  |  |  |  |  |  | **KRAS** | **Exon 2** | G12R |  |
| **BRAF** |  | V600E |  | **KRAS** | **Exon 4** | A146T |  | **KRAS** | **Exon 2** | G13C |  |
|  |  |  |  | **KRAS** | **Exon 4** | A146V |  | **KRAS** | **Exon 4** | K117N A>C |  |
|  |  |  |  | **KRAS** | **Exon 4** | A146P |  | **KRAS** | **Exon 4** | K117N A>T |  |
|  |  |  |  |  |  |  |  |  |  |  |  |
|  |  |  |  |  |  |  |  | **NRAS** | **Exon 2** | G12D |  |
|  |  |  |  |  |  |  |  | **NRAS** | **Exon 2** | G12C |  |
|  |  |  |  |  |  |  |  | **NRAS** | **Exon 2** | G12V |  |
|  |  |  |  |  |  |  |  | **NRAS** | **Exon 2** | G13D |  |
|  |  |  |  |  |  |  |  | **NRAS** | **Exon 2** | G13R |  |
|  |  |  |  |  |  |  |  |  |  |  |  |
|  |  |  |  |  |  |  |  | **NRAS** | **Exon 3** | Q61K |  |
|  |  |  |  |  |  |  |  | **NRAS** | **Exon 3** | Q61R |  |
|  |  |  |  |  |  |  |  | **NRAS** | **Exon 3** | Q61H |  |
|  |  |  |  |  |  |  |  | **NRAS** | **Exon 3** | Q61L |  |

**Table S2:**

| **Gene** | **Assay number** | **Assay length** | **CDS mutation** | **AA mutation** |
| --- | --- | --- | --- | --- |
| KRAS | dHsaCP2500586 | 57 | c.35G>C | p.G12A |
| KRAS | dHsaCP2500584 | 57 | c.34G>T | p.G12C |
| KRAS | dHsaCP2500596 | 57 | c.35G>A | p.G12D |
| KRAS | dHsaCP2500590 | 57 | c.34G>C | p.G12R |
| KRAS | dHsaCP2500588 | 57 | c.34G>A | p.G12S |
| KRAS | dHsaCP2500592 | 57 | c.35G>T | p.G12V |
| KRAS | dHsaCP2500594 | 57 | c.37G>T | p.G13C |
| KRAS | dHsaCP2500598 | 57 | c.38G>A | p.G13D |
| KRAS | dHsaCP2000133 | 61 | c.183A>C | p.Q61H |
| KRAS | dHsaCP2000131 | 61 | c.183A>T | p.Q61H |
| KRAS | dHsaCP2000101 | 61 | c.182A>T | p.Q61L |
| KRAS | dHsaCP2000135 | 61 | c.182A>G | p.Q61R |
| KRAS | custom made |  | c.351A>T | p.K117N/T |
| KRAS | custom made |  | c.351A>C | p.K117N/C |
| KRAS | dHsaCP2000079 | 80 | c.436G>A | p.A146T |
| KRAS | custom made | 80 | c.436G>C | p.A146P |
| KRAS | custom made | 80 | c.436A>T | p.A146V |
|  |  |  |  |  |
| BRAF | dHsaCP2000027 | 91 | c.1799T>A | p.V600E |
|  |  |  |  |  |
| NRAS | dHsaCP2500530 | 70 | c.34G>T | p.G12C |
| NRAS | dHsaCP2000095 | 70 | c.35G>A | p.G12D |
| NRAS | dHsaCP2500528 | 70 | c.35G>T | p.G12V |
| NRAS | dHsaCP2500526 | 70 | c.38G>A | p.G13D |
| NRAS | dHsaCP2500534 | 70 | c.37G>C | p.G13R |
| NRAS | dHsaCP2000065 | 65 | c.183A>T | p.Q61H |
| NRAS | dHsaCP2000067 | 65 | c.181C>A | p.Q61K |
| NRAS | dHsaCP2000069 | 65 | c.182A>T | p.Q61L |
| NRAS | dHsaCP2000071 | 65 | c.182A>G | p.Q61R |

**Table S3:** TTabelT

| **Mutation** | **Quantity** | **Frequency (%)** |
| --- | --- | --- |
| KRAS G12D | 37 | 12,59 |
| KRAS G12V | 26 | 8,84 |
| KRAS G13D | 22 | 7,48 |
| BRAF V600E | 76 | 25,85 |
| KRAS G12C | 8 | 2,72 |
| KRAS G12S | 4 | 1,36 |
| KRAS A146T | 4 | 1,36 |
| KRAS A146V | 1 | 0,34 |
| KRAS A146P | 1 | 0,34 |
| KRAS Q61R | 2 | 0,68 |
| KRAS Q61L | 1 | 0,34 |
| KRAS G12R | 1 | 0,34 |
| NRAS G12D | 2 | 0,68 |
| NRAS Q61K | 1 | 0,34 |
| NRAS Q61R | 2 | 0,68 |
| NRAS Q61L | 1 | 0,34 |

NOTE: Calculations based on the total number of patients (n=294).

**Table S4:**

| **Quartile** | **Mutated DNA (%)** | **Stage I (n)** | **Stage II (n)** | **Stage III (n)** |
| --- | --- | --- | --- | --- |
| **<25%** | <0.035 | 2 | 11 | 6 |
| **25-50%** | 0.035-0.098 | 1 | 12 | 8 |
| **50-75%** | 0.098-0.35 | 1 | 11 | 8 |
| **>75%** | >0.35 | 0 | 11 | 9 |

Percentage of mutated DNA ranged from 0.005-11.23%.
